# Role of Midkine in Cancer Drug Resistance: Regulators of Its Expression and Its Molecular Targeting

**DOI:** 10.3390/ijms24108739

**Published:** 2023-05-14

**Authors:** Minakshi Saikia, Nathan Cheung, Abhay Kumar Singh, Vaishali Kapoor

**Affiliations:** 1 Department of Radiation Oncology, Washington University in St. Louis School of Medicine, St. Louis, MO 63108, USA; msaikia@wustl.edu (M.S.); c.nathan@wustl.edu (N.C.); abhaysingh@wustl.edu (A.K.S.); 2 Siteman Cancer Center, St. Louis, MO 63108, USA

**Keywords:** midkine, drug resistance, cancer targeting, transcriptional regulators

## Abstract

Molecules involved in drug resistance can be targeted for better therapeutic efficacies. Research on midkine (MDK) has escalated in the last few decades, which affirms a positive correlation between disease progression and MDK expression in most cancers and indicates its association with multi-drug resistance in cancer. MDK, a secretory cytokine found in blood, can be exploited as a potent biomarker for the non-invasive detection of drug resistance expressed in various cancers and, thereby, can be targeted. We summarize the current information on the involvement of MDK in drug resistance, and transcriptional regulators of its expression and highlight its potential as a cancer therapeutic target.

## 1. Introduction

Midkine (MDK), encoded by the MDK gene, was first discovered through differential hybridization by examining the increased RNA levels of its cDNA in retinoic acid-induced differentiation in embryonal carcinoma cells [1]. This 13 kDa cysteine-rich protein consists of two main domains, each containing three antiparallel β-strands and multiple heparin-binding consensus sites, which, when bound to, prompt chemical and structural changes within the protein [2]. MDK is thus categorized as a heparin-binding protein, belonging to the family of heparin-binding growth associate molecules (HB-GAM), which contains one other protein 50% similar to MDK, pleiotrophin (PTN), both of which share identical functions [3].

**Figure 1:**
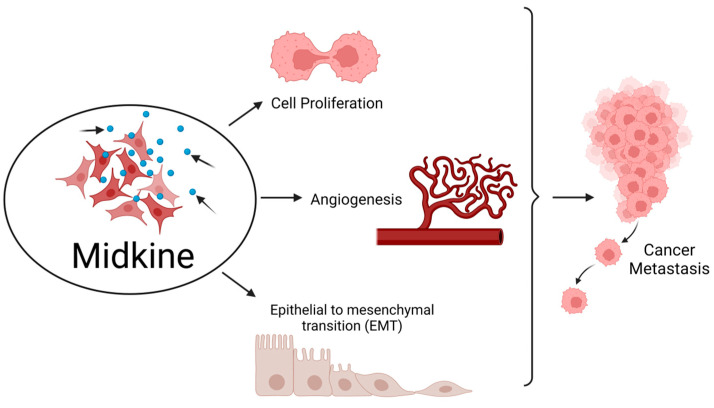
Midkine is involved in the hallmarks of cancer. MDK enhances cell proliferation and angiogenesis and also epithelial to mesenchymal transition (EMT), thereby aiding in metastasis of tumor from the primary site to distant locations. Created with BioRender.com.

MDK plays an essential role in developing and maintaining crucial systems, such as the nervous system. It is also known as a neurite growth-promoting factor as it helps in the development and survival of neurons [4]. Formerly, it was identified as developmentally vital as a retinoic acid-responsive gene product during mid-gestation, and hence it was named Mid-kine. Recent studies have highlighted the role of MDK as a biomarker due to its abnormally high expression in various malignancies, unlike in normal tissues with a weak or minimum expression. MDK mediates cell growth, survival, metastasis, and angiogenesis and accomplishes all the major hallmarks of cancer [5] (Figure 1).

MDK is a soluble growth factor secreted by the cells that produce it [6]. In the case of cancer, multiple sclerosis, ischemia, and other inflammation and neural diseases, MDK is a cytokine responsible for survival and proliferation in all of these conditions [7,8,9,10]. The presence of MDK in serum is associated with worse outcomes. When MDK is genetically silenced, it leads to a decrease in the proliferation of cancer cells [11]. While the exact pathways are not entirely known, the presence and association of MDK with carcinogenesis is evident [12]. 

MDK is overexpressed in at least 20 different cancers compared to normal levels in healthy individuals [6]. In pancreatic cancer cells, there was an increase in proliferation when MDK-depleted cells were exposed to MDK compared to the MDK-depleted cells without the treatment [13]. 

MDK overexpression can be correlated with the diagnosis and disease prognosis in various cancers, and it can be a promising molecular target in personalized medicine. Since it is a secretory cytokine and can be easily detected in body fluids such as blood, cerebrospinal fluid, urine, or tumor mRNA analysis, it is an inexpensive and convenient biomarker for the early diagnosis and prognosis, and also monitoring of the treatment response for NSCLC [6,14]. MDK is part of the diagnostic biomarker blood test that can detect early-stage lung cancer in at-risk populations [6]. 

Multi-drug resistance (MDR) impairs the clinical outcome of chemotherapeutics. Targeting MDK in non-small cell lung cancer (NSCLC) has demonstrated promising outcomes [5,14,15,16,17]. Many studies have reported MDK’s role in cancer progression and drug resistance [11,18,19,20,21,22,23]. Midkine is found to play a pivotal role in multi-drug resistance via different mechanisms such as inducing the overexpression of pro-proliferative molecules, inducing epithelial-mesenchymal transition (EMT), or others, such as increasing drug efflux pumps [22,24,25]. Inhibiting or downregulating MDK expression by using RNAi or siRNA shows drug sensitivity reverting to the resistant cells along with the enhancement in the efficacy of the chemotherapeutic agent [26,27]. However, not much is known about the regulation of MDK’s expression and function. In this review, we will specifically focus on the drug-resistance role of MDK and the transcriptional regulators of MDK expression that can aid in better targeting this protein to enhance the efficacies of the chemotherapeutics. 

## 2. MDK Drives Oncogenesis in Cancer via Various Pathways

**Figure 2:**
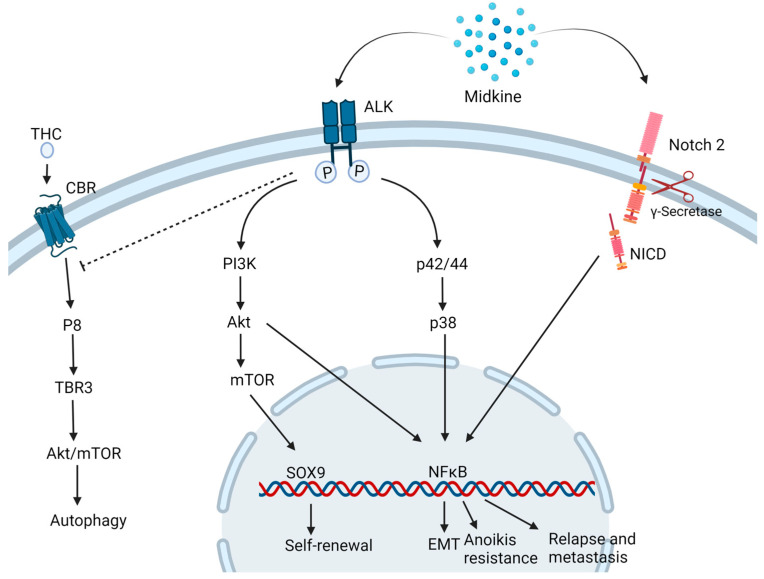
Midkine drives oncogenesis via different pathways. MDK binding to ALK receptor inhibits the autophagic cell death induced by Tetrahydrocannabinol (THC) and activates the PI3K/Akt/mTOR pathways inducing to self-renewal and stemness properties to the cells. Also, the MAPK pathway activates NFκB which triggers multiple downstream mechanisms of carcinogenesis such as epithelial to mesenchymal transition (EMT), Anoikis resistance and relapse and metastasis. MDK also binds to Notch 2 followed by the cleavage of Notch intracellular domain (NICD) by γ-secretase and activation of NFκB. CBR: Cannabinoid receptor. Created with BioRender.com.

MDK is found to drive oncogenesis by various pathways (Figure 2). 

In a neuroblastoma and osteosarcoma cells study, MDK induced cytoprotective function in the neighboring drug-sensitive cells against cell death induced by doxorubicin via the Akt pathway [19]. Additionally, in neuronal cell line PC12, MDK is found to impart neuroprotective functions via ERK activation [28]. In prostate cancer, MDK was found to be induced by TNF-α via the NFκB pathway. It activates the extracellular signal-regulated kinase ½ (Erk1/2) and p38 mitogen-activated protein kinase (MAPK) pathways in prostate cancer and LNCaP cells [29]. This cytokine is also linked to inducing EMT-mediated NF-κB activation upon interaction with the Notch 2 receptor [30] and angiogenesis, hence playing a key role in metastasis. MDK-Notch 2 signaling has also been shown to promote neuroblastoma through the downstream effector Hes-1 [31]. The binding of MDK to the anaplastic lymphoma kinase (ALK) receptors impedes the autophagic cell death-inducing activity of tetrahydrocannabinol (THC) which is the active component of marijuana, by preventing the upregulation of endoplasmic reticulum stress-related protein P8 and TRB3 [32]. MDK/ALK receptor axis is also noted to maintain the self-renewal capacity of the glioma-initiating cells (GICs) by preventing the autophagic degradation of the transcription factor SOX9 [33]. MDK activates mTOR by phosphorylating mTOR target RPS6 in uveal melanoma cells [34]. In hepatocellular carcinoma (HCC), MDK mediates anoikis resistance to the circulatory tumor cells (CTCs) and enhances tumor metastasis and relapse upon binding to ALK receptors through the PI3K/Akt/NFκB/TrkB axis, which makes a positive feedback loop [23].

## 3. MDK and Drug Resistance

**Figure 3:**
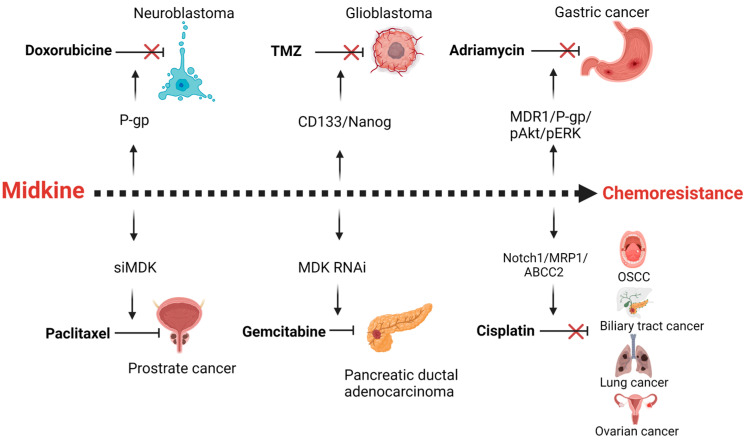
Midkine is a mediator of resistance of different chemotherapeutic agents in various cancers. MDK up-regulates p-glycoprotein (P-gp) in neuroblastoma cells in response to Doxorubicine chemotherapy. The Cancer stemness of the glioblastoma is enhanced by midkine upon temozolomide treatment. In gastric cancer adriamycin therapy results in the enhanced expression of MDK mediated multidrug resistance proteins. Cisplatin therapy fails in oral squamous cell carcinoma, biliary tract cancer, lung and ovarian cancer due to the expression of drug efflux pumps, mediated by MDK. Inhibition of MDK by siRNA or RNAi leads to increased sensitivity of paclitaxel and gemcitabine in prostrate and pancreatic ductal adenocarcinoma. Created with BioRender.com.

Drug resistance is the major obstacle in successful treatment of cancer. Resistance can be due to inherent mechanisms or acquired due to cancer cell adaptation to the chemotherapy stress. These mechanisms include an increase in efflux of the chemotherapeutic agents through the overexpression of drug transport proteins or activation of the DNA repair mechanisms, alterations in the drug target interactions, or alterations in the apoptotic response. MDK is found to be a putative gene involved in the drug resistance of various cancers (Figure 3).

One study completed in ESCC (esophageal squamous cell carcinoma) found that out of 66 ESCC samples studied using immunohistochemistry, MDK was over-expressed with a positive rate of 56.1% (37/66) [35]. Another group has shown the direct association of high expression of MDK with adverse OS in solid tumor patients by performing a meta-analysis on a total of 2097 patients from 17 observational studies [36]. Hu et al. have demonstrated in patient samples that intracellular Rhodamine123 accumulation in B cell lineage Acute lymphoblastic leukemia (ALL) cells is much lower than in normal cells, which positively correlates with the high MDK gene expression in those cells. They suggested that MDK might regulate drug efflux pumps [24]. In Pancreatic ductal adenocarcinoma (PDAC), as the dose of gemcitabine increased, so did the amount of MDK. When cells were depleted of MDK, they were no longer resistant to gemcitabine [30]. In melanomas, MDK effectively trains macrophages and cytotoxic T cells to prevent the immune system from attacking and recognizing the cancer cells [37]. Similarly, in a gastric cancer cell line, when MDK was silenced, there was a decrease in cell survival, indicating that a reduction of MDK might be involved in chemosensitization. Similarly, in HeLa cells, when MDK was overexpressed, there was a decrease in sensitivity to chemotherapeutic agents [38].

When human meningioma cells were treated with camptothecin, there was a decrease in active caspase-3 in the human meningioma cells, those that overexpressed MDK [39]. As an anti-apoptotic molecule, MDK prevents the natural defense system of the cell, further increasing cell growth and promoting MDK expression in tumor tissue [40]. MDK is also shown to impart cytoprotection to the Wilms’ tumor cells against cisplatin via the up-regulation of Bcl-2 proteins [41]. The role of MDK in inducing drug resistance towards different conventional chemotherapeutic agents in different cancer types is discussed in the following section.

### 3.1. Neuroblastoma

MDK is found to provide cytoprotective function in neuroblastoma. Analysis of both primary neuroblastoma tissues and cell lines reveals high expression of MDK mRNA [42]. Midkine levels were significantly higher (*p* < 0.0001) in neuroblastoma patients compared to non-tumor controls [43]. High plasma MDK levels correlated with poor disease prognosis [43]. Overexpression of MDK in neuroblastoma posed drug resistance towards doxorubicin and etoposide [18]. Doxorubicin-resistant (SK-N-SH/DoxR) neuroblastoma cells SK-N-SH were found to protect the neighboring drug-sensitive cells against doxorubicin via the secretory cytokine MDK. The multidrug-resistant gene P-Glycoprotein (P-gp) was found to be expressed in SK-N-SH cells that were doxorubicin-resistant, and knockdown of MDK using siRNA in SK-N-SH/DoxR cells reversed the sensitivity [44]. 

### 3.2. Glioblastoma

Glioblastoma (GBM) is an aggressive form of malignant glioma, and temozolomide (TMZ) is the primary agent for treating GBM. The mRNA expression of MDK in normal control samples was significantly lower (*p* < 0.05) than in GBM samples in The Cancer Genome Atlas (TCGA) and Genotype-Tissue Expression (GTEx) datasets [11]. MDK induces cell proliferation, cancer stemness, and invasion in glioma cells resulting in temozolomide (TMZ) resistance. Yu et al. have mechanistically established that TMZ resistance to MDK is due to the up-regulation of p-JNK through Notch 1, thereby elevating stemness marker expressions such as CD133 and Nanog [45]. 

### 3.3. Gastric Cancer

A group studied around 250 genes showing altered expression in 5-fluorouracil, cisplatin, or doxorubicin-resistant gastric cancer cell lines and found that MDK is overexpressed in all these cell types suggesting MDK as a multidrug-resistant gene [46]. Additionally, another study conducted in gastric cancer revealed that multi-drug resistance type1 (MDR1) and P-Glycoprotein were expressed in Adriamycin resistant cells and not in the Adriamycin sensitive cell. The same group has shown that MDK transfection of the Adriamycin sensitive cells resulted in resistance to the same via the phosphorylation of protein kinase B (Akt) and Extracellular signal-regulated protein kinase (ERK) [22]. The association of MDK levels in the serum and gastric premalignant lesions has been well studied and can be exploited as a diagnostic marker [47]. Serum midkine levels of gastric cancer patients were significantly higher (*p* = 0.02) than those of the healthy controls [48]. Additionally, cancer-associated fibroblasts (CAFs) mediate chemoresistance against cisplatin in gastric cancer cells via MDK, which up-regulates long non-coding RNA (LncRNA) suppressor of tumorigenicity seven antisense RNA1 (ST7-AS1) which activates PI3K/Akt pathway and promotes EMT [49].

### 3.4. Prostate Cancer

Immunohistochemistry of prostate cancer patient samples revealed that of the 80 clinical cancers examined, 69 specimens (86.3%) were immunoreactive for MDK, with metastatic lesions showing higher expression than the corresponding primary tumors [50]. Normal prostate tissues were negative or showed only weak staining. MDK expression is induced by cytokines and growth factors such as TNF-α, which support prostate cancer cell survival [29]. A combination of siRNA for MDK with the conventional drug paclitaxel showed promising enhancement in the anticancer effects of paclitaxel without any increase in drug dose or overall toxicity [27,51]. A study has also shown that silencing of MDK along with quercetin treatment can be a good approach to target prostate cancer stem cells (CSCs) [52].

### 3.5. Pancreatic Ductal Adenocarcinoma (PDAC)

PDAC had elevated levels of MDK, whereas normal pancreatic cells do not express MDK. In tissue microarray analysis of histological subtypes of pancreatic cancer, moderate to strong MDK expression was found in 36% (20 of 56) and 38% (21 of 56) of ductal adenocarcinomas, respectively [13]. Strong MDK expression was found in 64% (18 of 28) of papillary adenocarcinoma tissue samples as well as in 47% (9 of 19) of neuroendocrine patient samples [13]. A significant reduction in the proliferation of PDAC cells was observed when compared with control cells upon downregulation of MDK by siRNA or shRNA [13]. Gemcitabine is one of the frontline chemotherapeutics used to treat pancreatic cancer. A dose-dependent increase in MDK expression is reported in PNAC-1 and PaCA 5061, gemcitabine-treated resistant PDAC cells. In contrast, Gemcitabine-sensitive BxPC-3, L3.6p cells showed no up-regulation in MDK expression [30]. RNAi-mediated abrogation of MDK restored the chemosensitivity of the resistant cells towards gemcitabine. Alonso et al. blocked the ALK signaling pathway, which is one of the receptors for MDK binding, thereby inhibiting proliferation, self-renewal, tumorigenicity, and chemoresistance of the PDAC cells [26].

### 3.6. Biliary Tract Cancer

Biliary tract cancer (BTC) or cholangiocarcinoma is cancer in the duct that connects the liver, gall bladder, and small intestine. A significant association between high expression of MDK and clinically advanced tumor stage was found in BTC (T3/T4 vs. T1/T2; *p* = 0.007) [53]. In addition, disease-free survival was significantly lower (*p* < 0.001) in patients with positive MDK expression [53]. Cisplatin and gemcitabine are the frontline chemotherapeutics for BTC, but drug resistance impairs the overall survival in the patients. MDK induces epithelial-to-mesenchymal transition in the BLT cells via activating the NOTCH 1 signaling pathway [21].

### 3.7. Breast Cancer

Breast cancer is one of the leading causes of female cancer-related death cases. Plasma MDK in patients with breast cancer was found to be elevated compared to normal controls (ductal carcinoma in-situ, DCIS: *p* < 0.05; primary invasive cancer without distant metastasis: *p* < 0.001; metastatic disease: *p* < 0.00) [54]. Neoadjuvant chemotherapy (NCT) is the first line of treatment for locally advanced breast cancer (LABC) patients prior to surgery. However, poor pathological response to NCT is a common problem for treatable LBAC patients, and with time they may develop chemoresistance or metastasis, hence depriving them of proper treatment administration. Alteration of the non-coding RNA or miRNA is one of the strategies for chemoresistance adopted by the cells. A case study revealed miR-1275 was significantly lower in NCT-resistant patients, and MDK is a direct target of miR-1275. Low miR-1275 induced chemoresistance of breast cancer cells via MDK mediated PI3K/Akt Axis [55]. 

### 3.8. Lung Cancer

Serum MDK was found to be significantly overexpressed in patients with NSCLC compared to healthy controls (*p* < 0.001) [56]. Patients with high serum MDK concentration showed a significantly shorter overall survival compared to patients with low serum MDK expression (*p* < 0.05) [56]. EMT plays a prominent role in facilitating cancer progression via several mechanisms, such as drug resistance. Cells that are transiting from epithelial to mesenchymal phenotype show an increase in drug efflux pumps or anti-apoptotic molecules [57]. A study has shown that estradiol (E2) regulates EMT in lung adenocarcinoma via transcriptional activation of MDK [25]. Most stromal cells in cancer tissue comprise cancer-associated fibroblasts (CAF). These are found to secrete MDK, which promotes cisplatin resistance to Oral squamous cell carcinoma (OSCC), Ovarian and lung cancer cells by up-regulation of ATP binding cassette transporter (ABC) family proteins, multidrug-resistance associated protein-1 (MRP1) and ATP binding cassette subfamily C member two protein (ABCC2) via the long non-coding RNA (lncRNA) ANRIL, in tumor cells [20].

### 3.9. Ovarian Cancer

Epithelial ovarian cancer (EOC) is among the most common cancers for women worldwide, and the association of MDK with ovarian cancer has been studied for a long time [58]. Immunohistochemistry of EOC patient tissue samples showed that the expression of MDK significantly correlated with disease histology (*p* = 0.038) as well as differentiation grade (*p* < 0.001) [59]. Mirkin et al. have shown that cisplatin–resistant ovarian cancer cells express higher amounts of MDK than their drug-sensitive counterpart [19]. Big data-based identification and bioinformatics analysis have found the association of MDK as one of the 55 genes that are involved in the development, progression, and drug resistance of ovarian cancer [60]. 

## 4. Transcriptional Regulators of MDK Expression

**Figure 4:**
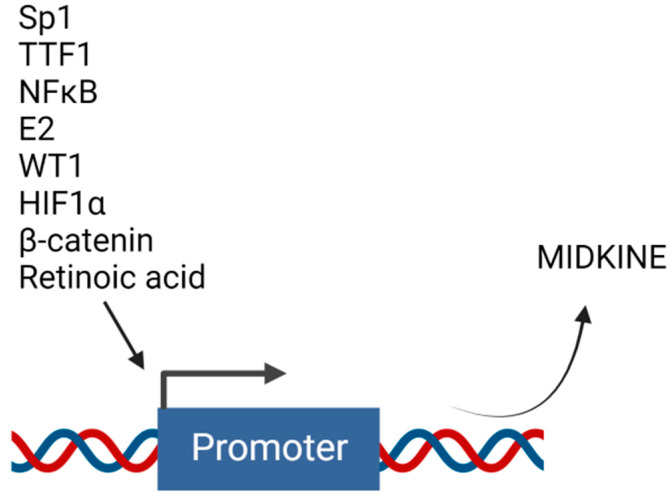
Upstream transcription factors that regulate midkine gene expression. Transcription of MDK can be induced by different molecules that act as transcription factors and bind to the promoter region of MDK. Created with BioRender.com.

Several studies have described the role of MDK in drug resistance. However, not much has been completed to elucidate the mechanisms of regulation of MDK’s expression. Transcriptional regulation is necessary for the production of any protein. The transcriptional machinery is controlled by transcription factors that bind to the gene’s promoter region to transcribe the respective gene product. MDK is found to be regulated by various transcriptional regulators (Figure 4). 

### 4.1. Estradiol

Estradiol (E2) is reported to elevate MDK mRNA expression in lung adenocarcinoma cells LTEP-a2 and A549 in a time-dependent manner. ICI 182,780 and tamoxifen, estrogen receptor (ER) inhibitors, inhibited the MDK expression induced by E2. In contrast, the antagonists for phosphoinositide-3 kinase and MAPK could not inhibit the same, suggesting the regulation of MDK by E2 at the transcriptional level. E2-induced Erβ was recruited to the estrogen response element in the MDK promoter. MDK regulates EMT, which plays a significant role in the migration of tumor cells in NSCLC. Knockdown of MDK showed a halt in EMT under E2 stimulation. MDK is an essential component of the E2-mediated EMT axis in NSCLC [25].

### 4.2. HIF-1α 

Hypoxia inside the tumor is a barrier that tumor tissue needs to mitigate to flourish inside the system. Two potential Hypoxia Response Elements (HREs) exist in the MDK gene promoter region. HIF-1α induces MDK expression during hypoxic conditions, increasing the vascularization of the small pulmonary arteries and paving the vasculature [61], providing the basic regulatory elements needed for the cancer cells to survive.

### 4.3. NF-κB

The promoter region of MDK has a functional binding site for nuclear factor-κB (NF-κB) [62]. This explains the overexpression of MDK during inflammatory conditions. In prostate cancer cells, TNF-α was found to up-regulate the NF-κB pathway, thereby inducing the transcription of MDK [29].

### 4.4. SP1

The transcription factor, specificity protein 1 is encoded by the gene SP1. This protein is essential during the embryonic and early postnatal period [63]. Similar to the altered expression of MDK during inflammation and cancer, expression of SP1 is also elevated in human glioma tissues more than in normal tissue. SP1 binds to the putative transcription site in the promoter region of MDK and directly results in the upregulation of the same. This interaction is found to have cooperated in the tumorigenesis and progression of glioma [64].

### 4.5. TTF-1

The thyroid transcription factor (TTF) -1 regulates lung parenchyma formation and gene expression. TTF-1 binds to the TTF regulatory elements present in the 5′-region of the MDK promoter, and MDK expression was absent in the lungs of TTF-null mice. It is observed that TTF-1 is required for pulmonary MDK expression but not for its expression in non-pulmonary tissues. Other transcription factors may control the regulation of MDK in other tissues [65]. 

### 4.6. Retinoic Acid

Retinoic acid is a metabolite of Vitamin A and is needed by the body for normal growth and development. Retinoic acid regulates transcription by interacting with nuclear retinoic acid receptors (RARs) that are bound to retinoic acid response elements (RARE) near the target genes [66]. The human MDK gene has an upstream site that responds to Retinoic acid [67]. 

### 4.7. Beta Catenin

Beta-catenin is the key player of the Wnt-mediated pathway involved in the cells’ proliferation, differentiation, and apoptosis. Knockdown of MDK substantially downregulates the carcinogenic properties exhibited by Beta-catenin, a multifunctional protein reported to act as a transcription factor. Beta-catenin has been shown to bind to the promoter region of MDK and regulate it transcriptionally [68].

### 4.8. WT1

Wilms’ tumor is an embryonal malignancy of the kidney, and MDK is expressed in high levels during mid-gestation for developmental regulation of the embryo. The tumor suppressor gene for Wilms’ tumor is WT1. A study has shown that all Wilms’ tumor samples studied have a high level of MDK, and there are two WT1 elements in the human MDK promoter region. They have suggested that MDK is a target gene of WT1 [69]. 

## 5. Strategies to Target Midkine

Since MDK is an important mediator of tumorigenesis, attempts have been made to target it for cancer therapy. Administration of MDK antisense oligodeoxynucleotide in nude mice bearing rectal carcinoma cells showed a much lower tumor burden [70]. Antisense oligonucleotide targeting MDK has also shown significant inhibition of hepatocellular carcinoma (HCC) [71] and enhanced the chemosensitivity of the HCC towards Adriamycin [72]. The same group has also demonstrated inhibition in the growth and proliferation of HCC, both in vitro and in vivo, upon treatment with antisense oligonucleotides of MDK loaded in nanoparticles [73]. Small interfering RNA (siRNA) targeting MDK could almost completely inhibit the MDK secretion in prostate cancer cells and enhance the cytotoxicity of paclitaxel-mediated chemotherapy [27]. The antitumor activity of cisplatin was augmented by the knockdown of MDK by siRNA in human gastric carcinoma cells [74]. 

A small molecule iMDK could suppress the expression of MDK, thereby retarding the growth and proliferation of the cells, probably by the retardation of the PI3K pathway [15]. Treating the primary effusion lymphoma (PEL) cells with iMDK resulted in strong induction of cell cycle arrest in the G2/M phase and led to the reduction of p-CDK1 protein level. This ultimately led to the activation of caspases and apoptosis of the PEL cells [75]. Administration of iMDK in the OSCC induced suppression of cluster of differentiation 31 (CD31) expression both in in vitro and in vivo OSCC models, inhibited cell proliferation, and also demonstrated inhibition of VEGF-mediated angiogenesis [76]. A group has used MDK RNA aptamers, demonstrating that it induced T regulatory cell expansion and prevented autoimmune diseases such as multiple sclerosis [77]. 

Antibody-based therapeutic strategies have also been adopted to target MDK. Monoclonal antibodies (mAb) have high binding specificity towards antigens on the cell surface and hence can be effectively exploited for the delivery of cytotoxic agents. Anti-MDK monoclonal antibody conjugated to doxorubicin proved to have a growth inhibitory effect in HCC cells HepG2 [78]. Similarly, an MDK-specific doxorubicin conjugated single-chain variable fragments (scFv) demonstrated the growth inhibitory potential of the antibody conjugate [79]. Growth inhibition of osteosarcoma cells was seen after treatment with functional anti-MDK antibody [80]. 

Low-density lipoprotein (LDL)-related-protein 1 (LRP1) is a receptor for MDK and induces oncogenic pathways after binding of MDK. A peptide derived from the LRP1, called MK-TRAP, binds with high affinity to MDK and prevents its binding to LRP1. As a result, the oncogenicity imparted by MDK and LRP1 signaling could be abrogated. Anti-MDK antibodies, as well as MK-TRAP, is also shown to suppress anchorage-independent growth of cancer cells [69]. Inhibition of MDK by anti-MDK antibody suppressed osteosarcoma cell proliferation and reduced tumor growth in vivo, and also significantly reduced lung metastasis of osteosarcoma [81]. 

Although successful preclinically, there are several challenges in the therapeutic targeting of MDK in humans using the strategies described above. siRNAs, being negatively charged, are membrane-impermeable. Due to their small size, they are rapidly cleared by the kidneys, limiting bioavailability [82]. Enzymatic degradation of siRNAs by serum endonucleases and RNAases is another major challenge in systemic delivery [82]. Local delivery to the site of action circumvents some of the problems, but further research is needed to successfully translate this strategy to the clinic. Similar to siRNAs, iMDK and peptides being small molecules, will face the challenge of renal clearance and poor bioavailability. Not much research has been completed on the specificity of iMDK or MK-TRAP. Nonspecific binding to other receptors on normal cells may lead to dose-limiting toxicities. Antibodies, on the other hand, have longer circulation time because of their larger size and re-circulation by neonatal receptor (FcRn) binding. The challenges with antibody-based therapeutics include high cost of production, limited tissue penetration, and pharmacokinetics.

## 6. Other Roles of MDK in Cancer

The secretory cytokine MDK is a low molecular weight protein with almost 50% pleiotrophin (PTN) homology. While both share similar functions [3], the association of MDK expression could be directly correlated with the stage of carcinogenesis [83]. MDK is present in the extracellular vesicles or exosomes secreted from PDAC cell lines and stimulates the malignant growth of neighboring non-cancerous cells [84]. This cytokine is not present in healthy adults but is fairly evident in the bloodstream from the onset of the malignancy, even before the development of other symptoms. This property of MDK is exploited as a biomarker to detect malignancy at inception. 

MDK is found to be interacting with immune cells, thereby modulating the same to play roles in resistance to chemotherapy or immunotherapy [85]. In gastric cancer cells, it is found that MDK induces the P38 signaling, thereby activating AP1 and increasing transcription of MHC class I chain-related proteins A and B (MICA/B). These are expressed in elevated levels during malignancy and inhibit natural killer cells’ cytotoxicity (NK), impairing the cells’ natural immune surveillance [86]. A recent study in HCC indicates that MDK might induce an immunosuppressive microenvironment in response to chemotherapeutic agents such as sorafenib. Sorafenib treatment enhances MDK expression, stimulating the accumulation of immunosuppressive myeloid-derived suppressor cells (MDSC) in the tumor microenvironment, further secreting IL-10. MDK inhibition enhanced the anti-PD-1 immunotherapy in HCC [87]. Interferons (IF) are a cytokine class with anti-proliferative, anti-viral, and immunomodulatory functions. One of these Ifs, IF-γ, is exploited to combat different types of cancer, but reports indicate that the same aids in metastasis; upon investigation was found that MDK is the response element of IF-γ and is responsible for IF-γ induced metastasis in different cancers [88]. MDK secretion by melanoma cells leads to an immune evasive microenvironment that favors immune suppression and cancer development in malignant melanoma [37]. 

Zhang et al. have demonstrated in NSCLC that CAF in the tumor microenvironment secretes MDK, which via the Wnt/β-catenin pathway up-regulates c-myc, thereby promoting glycolysis which aids in DNA damage repair. This ultimately results in the radioresistance of the NSCLC cells [89]. A group suggests that targeting MDK and P-gp can enhance the therapeutic efficacies of chemotherapeutic drugs [90].

MDK is identified to have a positive relation between serum biomarkers and histological biomarkers. High MDK levels could correlate with higher serum levels of cancer antigen (CA125) and carcinoembryonic antigen (CEA) in patients with EOC. Wu et al. demonstrated that MDK could be used to determine the clinicopathological parameters in EOC. This group showed that MDK levels could be used to assess the sensitivity of EOC towards paclitaxel and cisplatin chemotherapy, probably by inhibiting the expression of multi-drug resistance-associated protein 3 (MRP3) [59].

Metformin is a drug used in the treatment of type II diabetes, and it has been theorized as a potential anticancer drug specifically targeting the functions of MDK. Broadly it appears that most of the pathways and resulting detrimental effects of MDK may be inhibited by metformin, such as the PI3K and MAPK pathways. Metformin may counteract MDK in almost every way that contributes to MDK’s ability to contribute to cancer growth; hence metformin may act as a possible MDK inhibitor [91]. 

## 7. Conclusions

MDK is an emerging player in drug resistance in various cancers. Overall, blocking MDK-mediated drug resistance pathways may improve patient outcomes. Novel therapeutic strategies targeting MDK, either alone or in combination with the current standard of care, are needed for personalized medicine. Further research is needed to completely understand the role of MDK in driving carcinogenesis and therapeutic resistance.

## Data Availability

Not applicable.
